# Icariside II induces rapid phosphorylation of endothelial nitric oxide synthase via multiple signaling pathways

**DOI:** 10.7717/peerj.14192

**Published:** 2022-10-25

**Authors:** Wenpeng Song, Yiming Yuan, Xiaohui Tan, Yangyang Gu, Jianyu Zeng, Weidong Song, Zhongcheng Xin, Dong Fang, Ruili Guan

**Affiliations:** 1 Department of Urology, Peking University First Hospital, Beijing, China; 2 Institute of Urology, Peking University, Beijing, China; 3 Beijing Key Laboratory of Urogenital Diseases (male) Molecular Diagnosis and Treatment Center, Beijing, China; 4 Department of Dental Implant Center, Beijing Stomatological Hospital, School of Stomatology, Capital Medical University, Beijing, China; 5 Department of Radiation Medicine, Institute of Systems Biomedicine, School of Basic Medical Sciences, Peking University Health Science Center, Beijing, China

**Keywords:** Phosphorylation, Icariside II, Nitric oxide, Endothelial nitric oxide synthase, Flavonoids

## Abstract

Icariside II, as a favonoid compound derived from epimedium, has been proved to involed in a variety of biological and pharmacological effects such as anti-inflammatory, anti-osteoporosis, anti-oxidation, anti-aging, and anti-cancer but its mechanism is unclear, especially in terms of its effect on post-transcriptional modification of endothelial nitric oxide synthase (eNOS). Phosphorylation of eNOS plays an important role in the synthesis of nitric oxide in endothelial cells, which is closely related to erectile dysfunction, atherosclerosis, Alzheimer’s disease, and other diseases. Our study aims to investigate the effect and mechanism of Icariside II on the rapid phosphorylation of eNOS. In this study, human umbilical vein endothelial cells (HUVECs) were stimulated with Icariside II in the presence or absence of multiple inhibitors (1 µM), including LY294002 (PI3K-inhibitor), MK-2206 (AKT-inhibitor), Bisindolylmaleimide X (AMPK-inhibitor), H-89 (CaMKII-inhibitor), KN-62 (PKA-inhibitor), Dorsomorphin (PKC-inhibitor). The proliferation of HUVECs was assessed using cell counting kit-8 (CCK-8). The release of nitric oxide (NO) within HUVECs was detected via fluorescence probe (DAF-FM). Western blot was used to examine the effect of Icariside II on the expression of eNOS, phosphorylation of eNOS, and common signaling pathways proteins. In this study, Icariside II was found to promote the cell proliferation and rapid NO release in HUVECs. The phosphorylation of eNOS-Ser1177 was significantly increased after Icariside II stimulation and reached a peak at 10 min (*p* < 0.05). Meanwhile, the phosphorylation of eNOS-Thr495 was significantly decreased after 45 min of stimulation (*p* < 0.05). Following the intervention with multiple inhibitors, it was found that MK-2206 (AKT inhibitor), LY294002 (PI3K inhibitor), KN-62 (AMPK inhibitor), and Bisindolylmaleimide X (PKC inhibitor) could significantly inhibit the phosphorylation of eNOS-Ser1177 caused by Icariside II (*p* < 0.05), while MK-2206, LY294002, and Bisindolylmaleimide X reversed the alleviated phosphorylation of eNOS-Thr495. We concluded that Icariside can regulate rapid phosphorylation of eNOS- Ser1177 and eNOS-Thr495 via multiple signaling pathways, resulting in the up-regulation of eNOS and the increased release of NO.

## Introduction

The endothelium consists of a single layer of specialized cells (endothelial cells) that form the interface between the vascular lumen and smooth muscle cells (Cyr, Huckaby & Zuckerbraun, 2020). In the past, the vascular endothelium was thought to function only as a mechanical barrier. However, it has been determined that the endothelium is a tissue that regulates vascular tone, cell growth, and interactions between blood cells and vessel walls (Bhang et al., 2018; Godo et al., 2016). It also synthesizes various growth factors and vasoactive substances, and responds to the physical and chemical signals (Signorello et al., 2011; Cheng et al., 2019; Garcia et al., 2017; Busse & Fleming, 1998). Endothelial cells are remarkably plastic according to their environment, which regulates specific organ development and maintains normal organ homeostasis by producing tissue-specific secretions (Song et al., 2021; Rafii et al., 1995; Raynaud et al., 2013). Meanwhile, endothelial cells, in turn, share a common set of functions, including hemostasis, maintenance of vascular permeability, mediation of acute and chronic immune responses to various injuries, and control of vascular tone (Cyr, Huckaby & Zuckerbraun, 2020). Endothelial dysfunction is characterized by reduced nitric oxide (NO) synthesis and NO sensitivity since NO produced by endothelial cells is a pivotal regulator of endothelial function in balance (Donato et al., 2011). NO is a strong vasodilator and anti-inflammatory signaling molecule, which plays a key role in maintaining vasodilator and vasoconstriction, inhibiting smooth muscle cell migration and proliferation, holding the balance between fibrinolysis and thrombosis, and regulating adhesion and aggregation of platelet (Shi & Vanhoutte, 2017; Popyhova et al., 2020; Konukoglu & Uzun, 2017; Heeringa et al., 2000; Jones et al., 1999). NO is also able to promote angiogenesis by up-regulating the levels of vascular endothelial growth factor (VEGF) and vascular endothelial growth factor receptor-2 (VEGFR-2) *in vivo* and *in vitro* (Milkiewicz et al., 2005; Hebert, Siavash & Sauk, 2005).

*In vivo*, endothelial cells can regulate NO synthesis by activating endothelial nitric oxide synthase (eNOS). eNOS is mainly regulated by protein interaction and multi-site phosphorylation, in which the phosphorylation state of the enzyme-specific serine, threonine, and tyrosine residues significantly affects eNOS activity (Kolluru, Siamwala & Chatterjee, 2010). So far, several phosphorylation residues have been proved to be related to eNOS activity, including Ser113, Thr495, Ser615, Ser633, and Ser1177. The most thoroughly studied residues are activation of eNOS-Ser1177 and inhibition of eNOS-Thr495 (Chen et al., 1999; Heiss & Dirsch, 2014). Although a large number of studies on eNOS phosphorylation have been published in recent decades, the specific molecular mechanisms have not been fully understood. Multiple protein kinases, including AMPK, CaMKII, PKA, PKC, PI3K, ERK, CHK1, and CDK5, have been indicated to constitute the complex regulatory network of eNOS phosphorylation (Heiss & Dirsch, 2014; Wu et al., 2022; Lee et al., 2021; Lee et al., 2018; Xing et al., 2015). Changes in the phosphorylation status of eNOS have an impact on a large number of disease processes including atherosclerosis, hyperhomocysteine, myocardial infarction, reperfusion injury, cerebral ischemia, and erectile dysfunction (Kolluru, Siamwala & Chatterjee, 2010). Overall, the regulation of eNOS phosphorylation is of great significance for the understanding of endothelial dysfunction.

Epimedium is traditional herbal medicine and functional food commonly used in Asia, which can be used to treat and prevent various diseases such as erectile dysfunction, osteoporosis, and depression (He, Wang & Shi, 2020). Icariin and Icariside II derived from epimedium belong to flavonoids and have a variety of biological and pharmacological effects such as anti-inflammatory, anti-osteoporosis, anti-oxidation, anti-aging, and anti-cancer (Xu et al., 2021; Liu et al., 2011; Khan et al., 2015). Liu et al. (2011) found that Icariside II can up-regulate eNOS expression and improve vascular endothelial function by activating EGF/EGFR signaling pathway in porcine arterial endothelial cells. This positive effect of Icariside II was also found in the human umbilical vein endothelial cells (HUVECs) (Tan et al., 2021). Another study indicated that Icariside II is able to promote the proliferation of cavernous endothelial cells and eNOS-Ser1177 phosphorylation by up-regulating ERK1/2 and AKT signaling pathways, alleviating the endothelial cell damage caused by high glucose conditions (Li et al., 2015). However, the effect of Icariside II on rapid phosphorylation of eNOS in endothelial cells has not been fully investigated. In this study, we investigated the rapid regulation of Icariside II on common phosphorylation residues of eNOS and explored its potential mechanisms.

## Materials & Methods

### Cells culture

HUVECs (Catalog #8000; Sciencell, Carlsbad, CA, USA) were purchased from ScienCell Research Laboratories and cultured in endothelial cell medium (ECM, Catalog #1001; Sciencell, Carlsbad, CA, USA), supplemented with 5% fetal bovine serum (FBS, Catalog #0025; Sciencell, Carlsbad, CA, USA), 1% endothelial cell growth supplement (Catalog #1052; Sciencell, Carlsbad, CA, USA), 100 U/ml of penicillin and 100 ug/ml streptomycin solution (Catalog #0503; Sciencell, Carlsbad, CA, USA). HUVECs were incubated at 37 °C with an atmosphere of 5% CO_2_ in the humidified incubator (Forma 3110; ThermoFisher Scientific, Lincoln, NE, USA) and passages 3–5 were served for subsequent experiments.

HUVECs were serum-starved in ECM without fetal bovine serum for 4 h before treatment of Icariside II and inhibitors were added. Cells were treated for 0.5, 1, 3, 5, 10, 15, 30, 45 or 60 min with 10^−5^, 10^−6^, 10^−7^, 10^−8^ or 10^−9^ M Icariside II with or without addition of the PI3K inhibitor LY294002 (1 mM; Catalog #HY-10108; MedChemExpress, NJ, USA), AKT inhibitor MK-2206 (1 mM; Catalog #HY-10358; MedChemExpress), AMPK inhibitor Dorsomorphin (1 mM; Catalog #HY-13418; MedChemExpress), CaMKII inhibitor KN-62 (1 mM; Catalog #HY-13290; MedChemExpress), PKA inhibitor H-89 (1 mM; Catalog #HY-15979A; MedChemExpress) or PKC inhibitor Bisindolylmaleimide X (1 mM; Catalog #HY-108136A, MedChemExpress) (Table 1).

**Table 1:** Inhibitors applied in this study.

Target kinases	Inhibitors
AKT	MK-2206
PI3K	LY294002
AMPK	Dorsomorphin
CaMKII	KN-62
PKA	H-89
PKC	Bisindolylmaleimide X

**Notes.**

Abbreviation AKT Protein kinase B PI3K Phosphatidylinositol-3-kinase AMPK AMP-activated protein kinase CaMKII Calcium-CaM-dependent protein kinase II PKA Protein kinase A PKC Protein kinase C

### Western blot

After being stimulated with Icariside II and inhibitors, HUVECs were washed with cold PBS (Catalog #SH30256.01; Hyclone, UT, USA). Total protein was extracted from the cells using lysis buffer containing RIPA (Strong) (#KGP702; Keygen Biotech, Nanjing, China), 1 mM Phenylmethylsulfonyl fluoride (Catalog #KGP610; Keygen Biotech), 1X protease inhibitor (Catalog #KGP603; Keygen Biotech) and 1X Phosphatase inhibitor (Catalog #KGP602; Keygen Biotech). Then the lysates were boiled with 5X SDS-PAGE loading buffer (Catalog #P1040, Solarbio, Beijing, China) for 8 min.

The samples containing 20 µg of protein were electrophoresed in 10% polyacrylamide gel and transferred to a polyvinylidene fluoride membrane. After being blocked for 1 h at room temperature, the membrane was incubated at 4 °C overnight with primary antibodies to p-eNOS^Ser1177^ (1:300; Catalog #9571; Cell Signaling Technology, Danvers, USA), p-eNOS^Thr495^ (1:300; Catalog #9574; Cell Signaling Technology), p-eNOS^Ser113^ (1:300; Catalog #9575; Cell Signaling Technology), p-PI3 Kinase p85^Tyr458^/p55^Tyr199^ (1:300; Catalog #17366; Cell Signaling Technology), p-AKT^ser473^(1:1000; Catalog #4060; Cell Signaling Technology), p-PKC*α*/*β*II^Thr638/641^ (1:300; Catalog #9375; Cell Signaling Technology), p-AMPK *α*^Thr172^ (1:300; Catalog #2535; Cell Signaling Technology), eNOS (1:300; Catalog #A1548; ABclonal, Woburn, MA, USA) and *β*-Actin (1:20000; Catalog #10205-2-AP; Proteintech, Rosemont, IL, USA).

After incubated with secondary antibodies, the images of membranes’ signals were obtained by using the Syngene G-Box imaging system (Syngene, Cambridge, UK) via ECL Plus Western Blotting Substrate (Catalog #32132; ThermoFisher Scientific).

### Nitric oxide release measurement

According to the manufacturer’s protocol, HUVECs were seeded into 96-well plates. At the confluence of 80%, cells were treated with 5 µM NO diacetate 3-Amino,4-aminomethyl-2′, 7′-difluorescein (DAF-FM DA; Catalog #s0019; Beyotime Biotech, Shanghai, China) for 30 min in serum-free medium, followed by drug stimulating with Icariside II for 0–60 min at 37 °C. Control groups were added with an equal volume of serum-free medium. After washing 3 times with PBS, the fluorescence images were collected by fluorescence microscope with excitation at 495 nM and emission at 515 nM (DMI 6000B, Leica Microsystems, Nussloch, Germany). Fluorescence images were analyzed with ImagePro Plus software (version 6.0, Media Cybernetics Inc, Bethesda, MD, USA) for calculating the mean density.

### Cell proliferation and cytotoxicity assay

Cell Counting Kit-8 (Catalog #CK04; Dojindo Molecular Technologies, Kumamoto, Japan) and the manufacturer’s protocol were applied for cell proliferation and cytotoxicity assay. After being dispensed in 96-well plates for 12 h, HUVECs were treated with Icariside II in various concentrations (0, 10^−5^, 10^−6^, and 10^−7^ M) for 24 and 48 h. After two washes with ECM, 10 µL CCK-8 solution was added to each well and incubated for 1 h in the incubator. Then, the microplate reader (Catalog #51118170; Thermo Fisher Scientific) was performed to measure the absorbance of each well at 450 nm.

### Statistical analysis

All experiments were repeated at least three times. All data were analyzed using GraphPad Prism, version 9.0 (GraphPad Software, San Diego, CA, USA) and shown as mean ± standard error of the mean (SEM). One-way ANOVA analysis was used for comparison between different groups. Statistical significance was considered when *P*-values were less than 0.05.

## Results

### Icariside II promoted the proliferation of HUVECs

**Figure 1:**
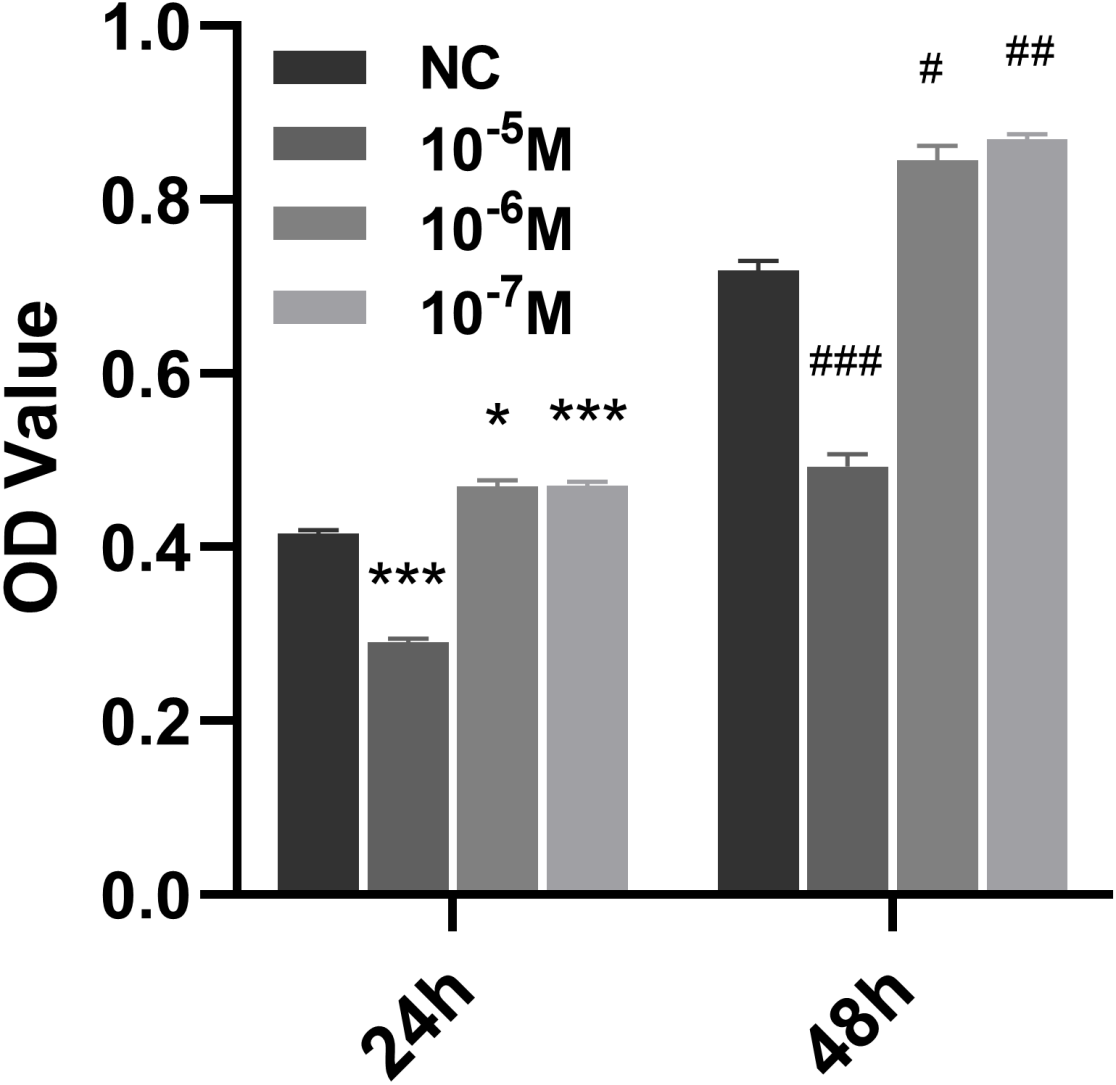
Effect of Icariside II on the proliferation of HUVECs. CCK8 kit was used to detect the proliferation of HUVECs at different concentrations of Icariside II (10^−5^M, 10^−6^M, 10^−7^M). (One-way ANOVA:^∗^ *p* < 0.05,^∗∗^ *p* < 0.01,^∗∗∗^ *p* < 0.001, Icariside II treated for 24 h vs NC, # *p* < 0.05, ## *p* < 0.01, ### *p* < 0.001, Icariside II treated for 48 h vs NC). Results are expressed as the mean ±SEM analyzed from three independent experiments.

To study the effect of Icariside II on the proliferation of HUVECs, a stimulation of Icariside II for 24 and 48 h was used, showing that the proliferation of HUVECs was significantly promoted at the concentration of 10^−6^ and 10^−7^ M. In contrast, 10^−5^ M of Icariside II showed a significant detraction effect (Figs. 1A, 1B). Therefore, Icariside II with a concentration below 10^−5^ was used for subsequent assays.

### Icariside II rapidly increased NO release

**Figure 2:**
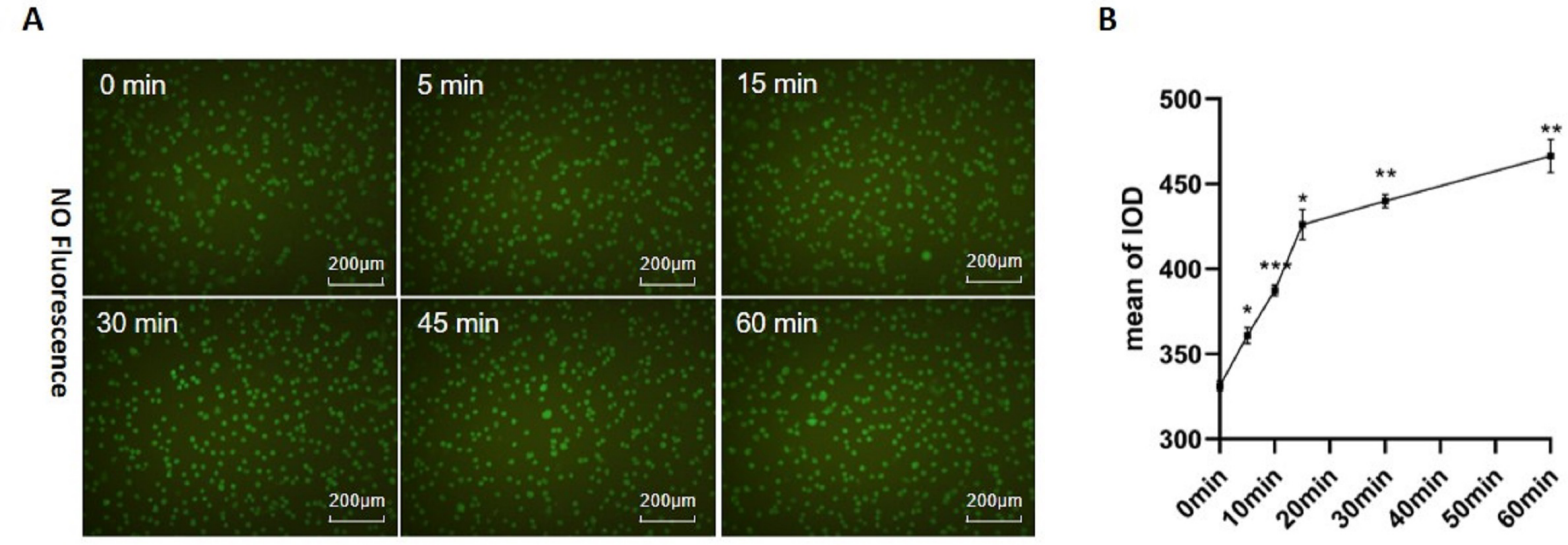
Effect of Icariside II stimulation on NO release. (A) HUVECs were stimulated with Icariside II (1*10^−6^M) for 5, 15, 30, 45, and 60 min and loaded with DAF-FM DA. Fluorescence imaging was performed to detect intracellular NO release. (B) Quantitative analysis of NO release. (One-way ANOVA: ^∗^*p* < 0.05,^∗∗^ *p* < 0.01, ^∗∗∗^ *p* < 0.001, experimental groups vs NC). Results are shown as one representative image and as the mean ± SEM of quantified data from three independent experiments.

In this study, the NO probe (DAF-FM DA) was adopted to detect the effect of Icariside II (10^−6^M) on NO release. NO release of HUVECs significantly increased after Icariside II was stimulated for 5 min. Within one hour, the mean signals increased gradually with the stimulation time, which indicated that Icariside II could up-regulate NO release of HUVECs rapidly (Figs. 2A, 2B).

### Icariside II stimulation did not alter total eNOS expression

**Figure 3:**
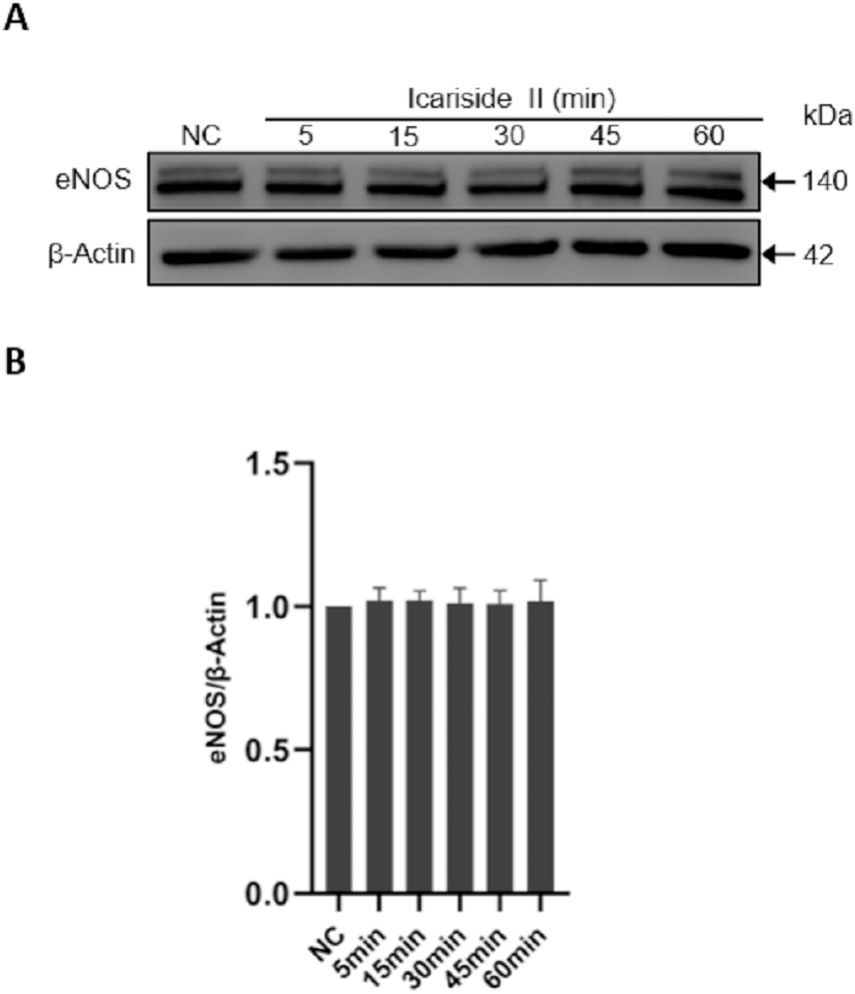
Effect of Icariside II on total eNOS expression. HUVECs were stimulated with Icariside II (1 µM) for 5, 15, 30, 45, and 60 min. (A) Western blot analysis of the expression of total eNOS. (B) Quantitative analysis of total eNOS expression. (One-way ANOVA:^∗^ *p* < 0.05, ^∗∗^ *p* < 0.01,^∗∗∗^ *p* < 0.001, experimental groups vs NC). Results are shown as one representative blot and as the mean ± SEM of quantified data from three independent experiments.

To investigate the effect of Icariside II on the expression of total eNOS, Icariside II (10^−6^ M) was used to stimulate HUVECs for 0, 5, 10, 15, 30, and 60 min. No significant changes were identified in Icariside II-treated groups compared with controls, suggesting that Icariside II did not affect the total eNOS expression of HUVECs within 60 min. (Figs. 3A, 3B).

### Icariside II rapidly induced eNOS-Ser1177 phosphorylation of HUVECs via PI3K/AKT, AMPK, and PKC signaling pathway

**Figure 4:**
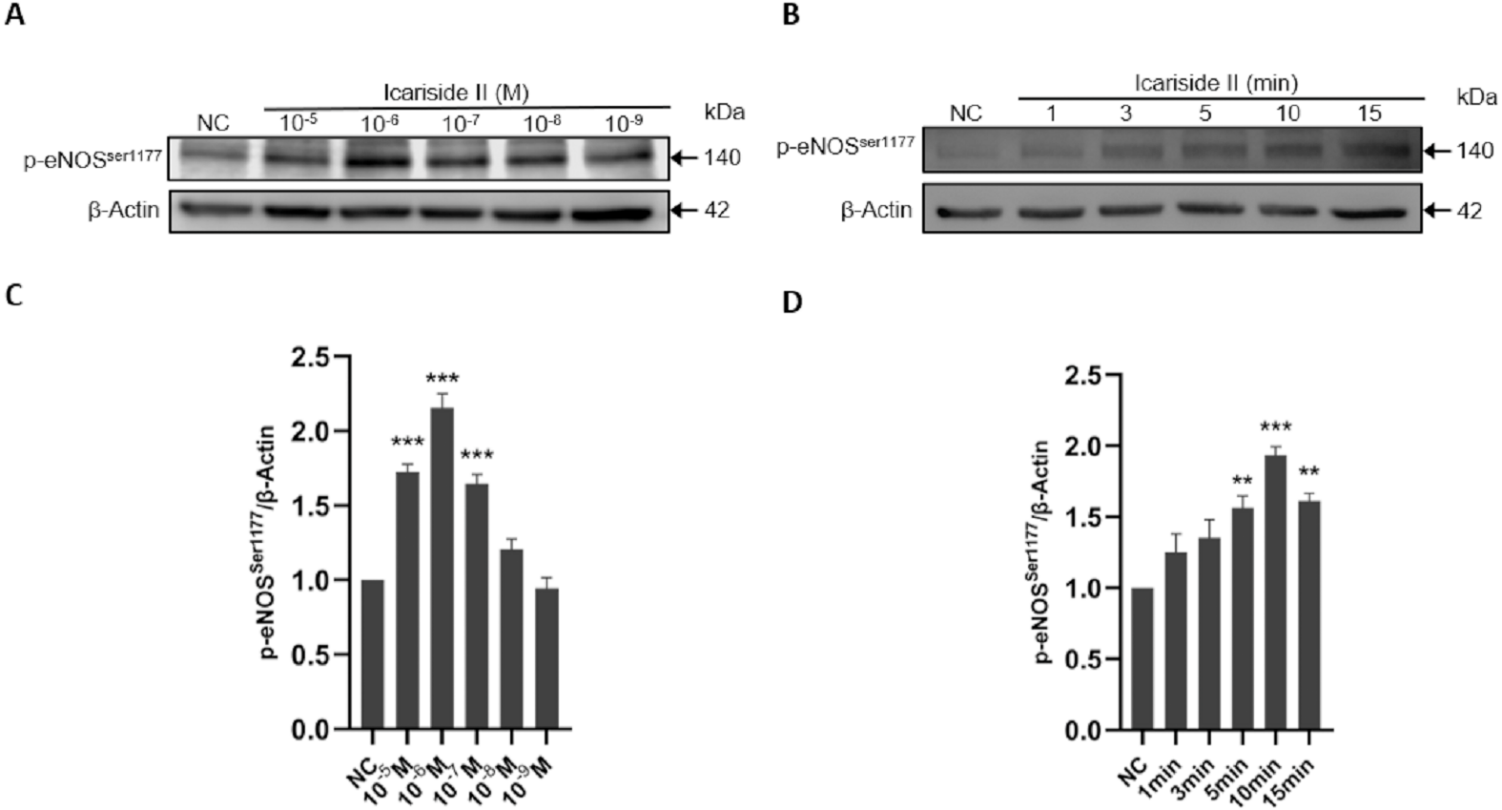
Effect of Icariside II on the dose- and time-dependent expression of p-eNOS^Ser1177^. HUVECs were stimulated with Icariside II (10^−5^M, 10^−6^M, 10^−7^M, 10^−8^M, 10^−9^M) for 1, 3, 5, 10, 15 min. (A) Western blot analysis of the expression of p-eNOS^Ser1177^ stimulated by different concentrations and time. (B) Quantitative analysis of total eNOS expression. (One-way ANOVA:^∗^ *p* < 0.05, ^∗∗^ *p* < 0.01,^∗∗∗^ *p* < 0.001, experimental groups vs NC). Results are shown as one representative blot and as the mean ± SEM of quantified data from three independent experiments.

To further clarify the experimental concentration of Icariside II in subsequent experiments, HUVECs were treated with different concentrations of Icariside II (10^−5^ M, 10^−6^ M, 10^−7^ M, 10^−8^ M, 10^−9^ M). It showed that Icariside II increased the phosphorylation of eNOS-Ser1177 in a dose-dependent manner, and reached the peak at the concentration of 10^−6^ M. On the other hand, the level of p-eNOS^Ser1177^ in 10^−8^ M and 10^−9^ M Icariside II-treated groups were not significantly different from that in the normal control group (Figs. 4A, 4C). Combined with the results of CCK-8, 10^−6^ M Icariside II was selected as the experimental concentration for subsequent experiments in this study.

To determine the optimal time-point for eNOS-Ser1177 phosphorylation, 0, 1, 3, 5, 10, and 15 min were selected as the stimulation time of Icariside II. The results showed that the phosphorylation of eNOS- Ser1177 was significantly increased only 5 min after the treatment of Icariside II. Furthermore, the phosphorylation level of eNOS-Ser1177 gradually increased and reached a peak at 10 min (Figs. 4B, 4D). Therefore, 10 min was selected as the experimental time-point for subsequent experiments in this study.

**Figure 5:**
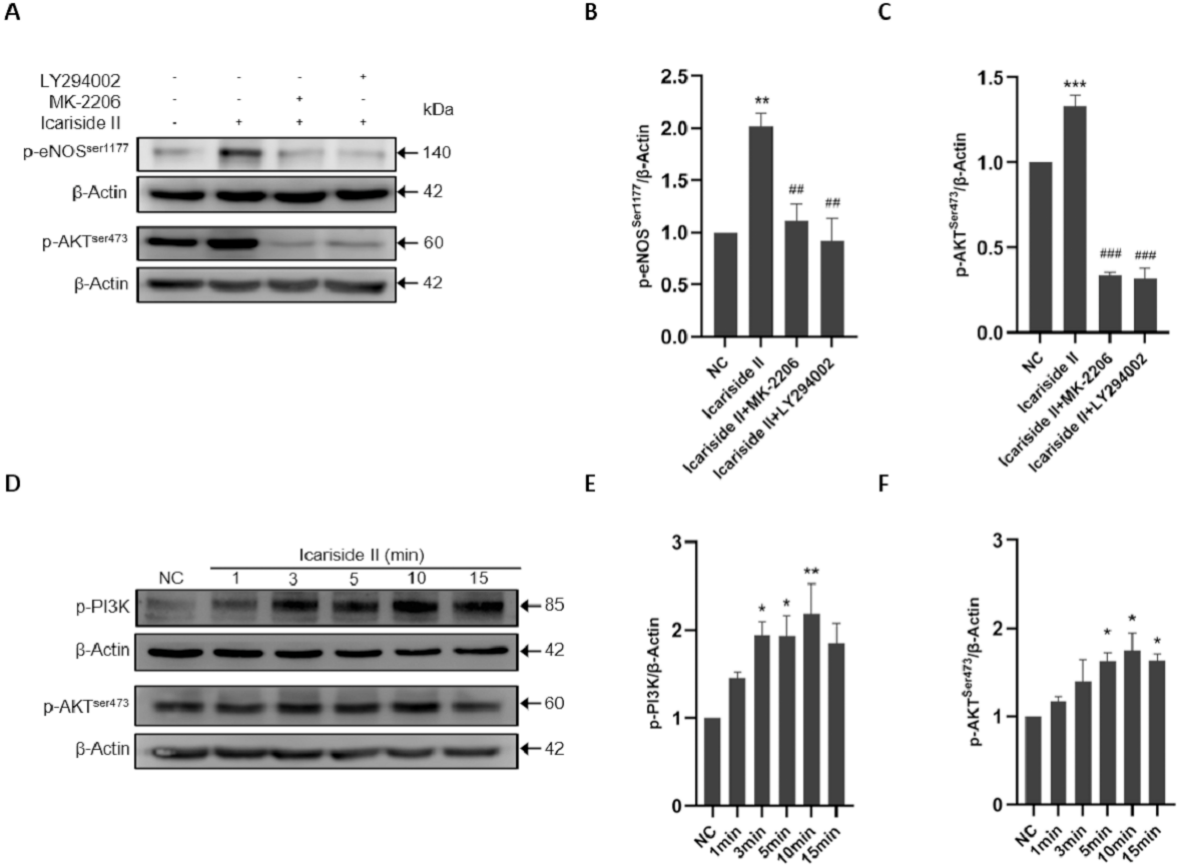
Icariside II regulate the expression of p-eNOS^Ser1177^ via PI3K/AKT signaling pathway. (A) Icariside II-induced eNOS-Ser1177 and AKT-Ser473 phosphorylation were abrogated by PI3K and AKT inhibitors (LY294002 and MK-2206) at 10 min. (B, C) Quantitative analysis of p-eNOS^Ser1177^ and p-AKT^Ser473^ expression. (One-way ANOVA:^∗∗^ *p* < 0.01,^∗∗∗^ *p* < 0.001, experimental groups vs NC, ## *p* < 0.01, ### *p* < 0.001, experimental groups vs Icariside II group). (D) Western blot analysis of the expression of p-PI3K and p-AKT^Ser473^ stimulated by Icariside II. (D, E) Quantitative analysis of p-PI3K and p-AKT^Ser473^ expression. (One-way ANOVA:^∗^ *p* < 0.05,^∗∗^ *p* < 0.01, experimental groups vs NC). Results are shown as one representative blot and as the mean ± SEM of quantified data from three independent experiments.

**Figure 6:**
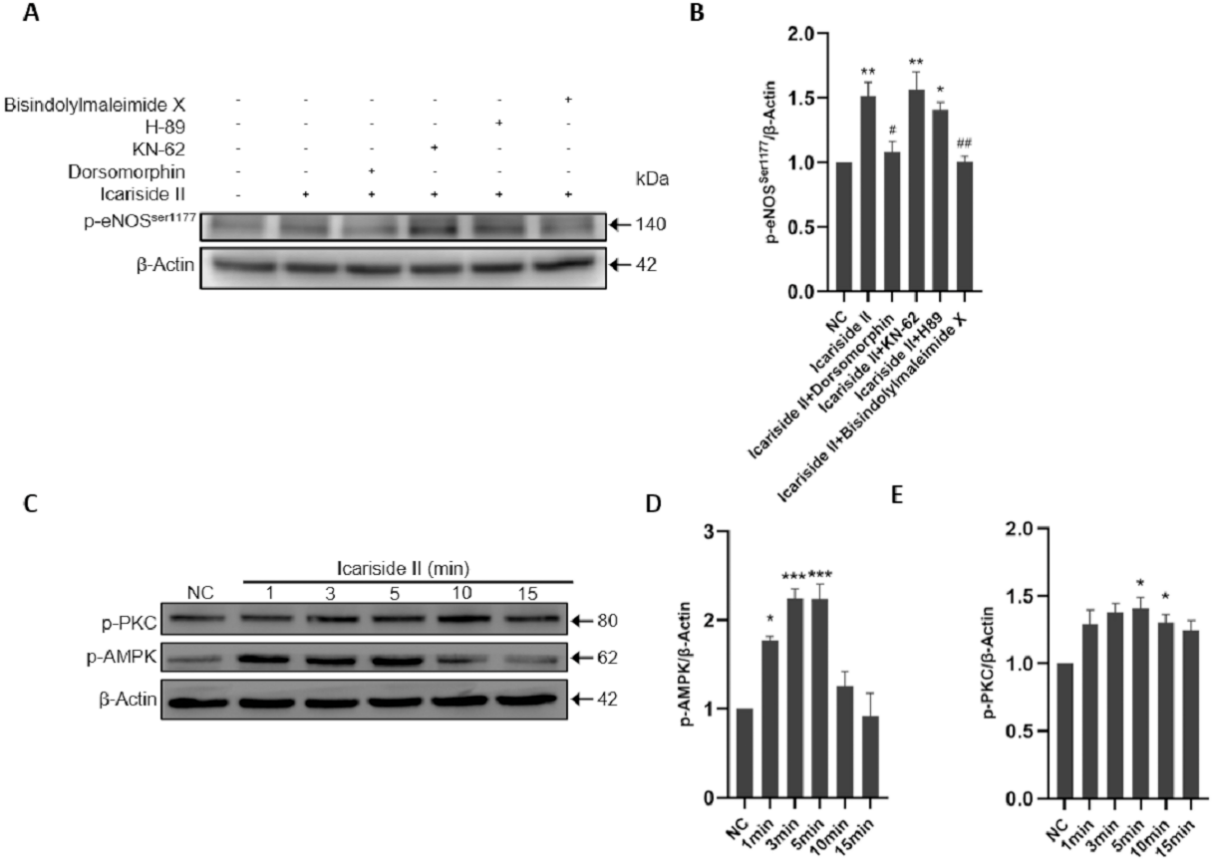
Icariside II regulate the expression of p-eNOS^Ser1177^ via AMPK and PKC signaling pathway. (A) Icariside II-induced eNOS-Ser1177 phosphorylation was abrogated by AMPK and PKC inhibitors (Bisindolylmaleimide X and Dorsomorphin), while CaMKII and PKA inhibitors (H-89 and KN-62) were not affected. (B) Quantitative analysis of p-eNOS^Ser1177^ expression. (One-way ANOVA:^∗^ *p* < 0.05,^∗∗^ *p* < 0.01, experimental groups vs NC, # *p* < 0.05, ## *p* < 0.01, experimental groups vs Icariside II group). (C) Western blot analysis of the expression of p-PI3K and p-AKT^Ser473^ stimulated by Icariside II. (D, E) Quantitative analysis of p-AMPK and p-PKC expression. (One-way ANOVA:^∗^ *p* < 0.05,^∗∗∗^ *p* < 0.001, experimental groups vs NC). Results are shown as one representative blot and as the mean ± SEM of quantified data from three independent experiments.

To clarify the specific mechanism of eNOS-Ser1177 phosphorylation regualted via Icariside II, we explored the upstream signaling pathways such as PI3K/AKT/eNOS. The p-eNOS ^Ser1177^ and p-eNOS^Thr495^ expression were significantly increased after Icariside II stimulation for 10 min, which was reversed by the use of PI3K inhibitor (LY294002) and AKT inhibitor (MK-2206) (Figs. 5A–5C). After stimulation of HUVECs with Icariside II (0, 1, 3, 5, 10, and 15 min), the expression of p-PI3K and p-AKT^Ser473^ were significantly upregulated and peaked at 10 min (Figs. 5D–5F). Then, AMPK, CaMKII, PKA, and PKC signaling pathways were also explored. The expression of p-eNOS^Ser1177^ was significantly increased in HUVECs treated with Icariside II for 10 min. The up-regulation was yet alleviated when treated with AMPK inhibitor (Dorsomorphin) and PKC inhibitor (Bisindolylmaleimide X). The phosphorylation of eNOS-Ser1177 in the Icariside II+CaMKII inhibitor (KN-62) group and the Icariside II+PKA inhibitor (H-89) group had no significant differences from Icariside II group (Figs. 6A, 6B). After treated with Icariside II alone (0, 1, 3, 5, 10, and 15 min), the expression of p-AMPK and p-PKC were significantly increased, in which p-AMPK peaked at 3 min and p-PKC peaked at 5 min (Figs. 6C–6E). These results suggested that Icariside II rapidly regulated the phosphorylation of eNOS-Ser1177 by activating PI3K/AKT, AMPK, and PKC signaling pathways.

### Icariside II rapidly induced eNOS-Thr495 dephosphorylation of HUVECs via PI3K/AKT and PKC signaling pathway

**Figure 7:**
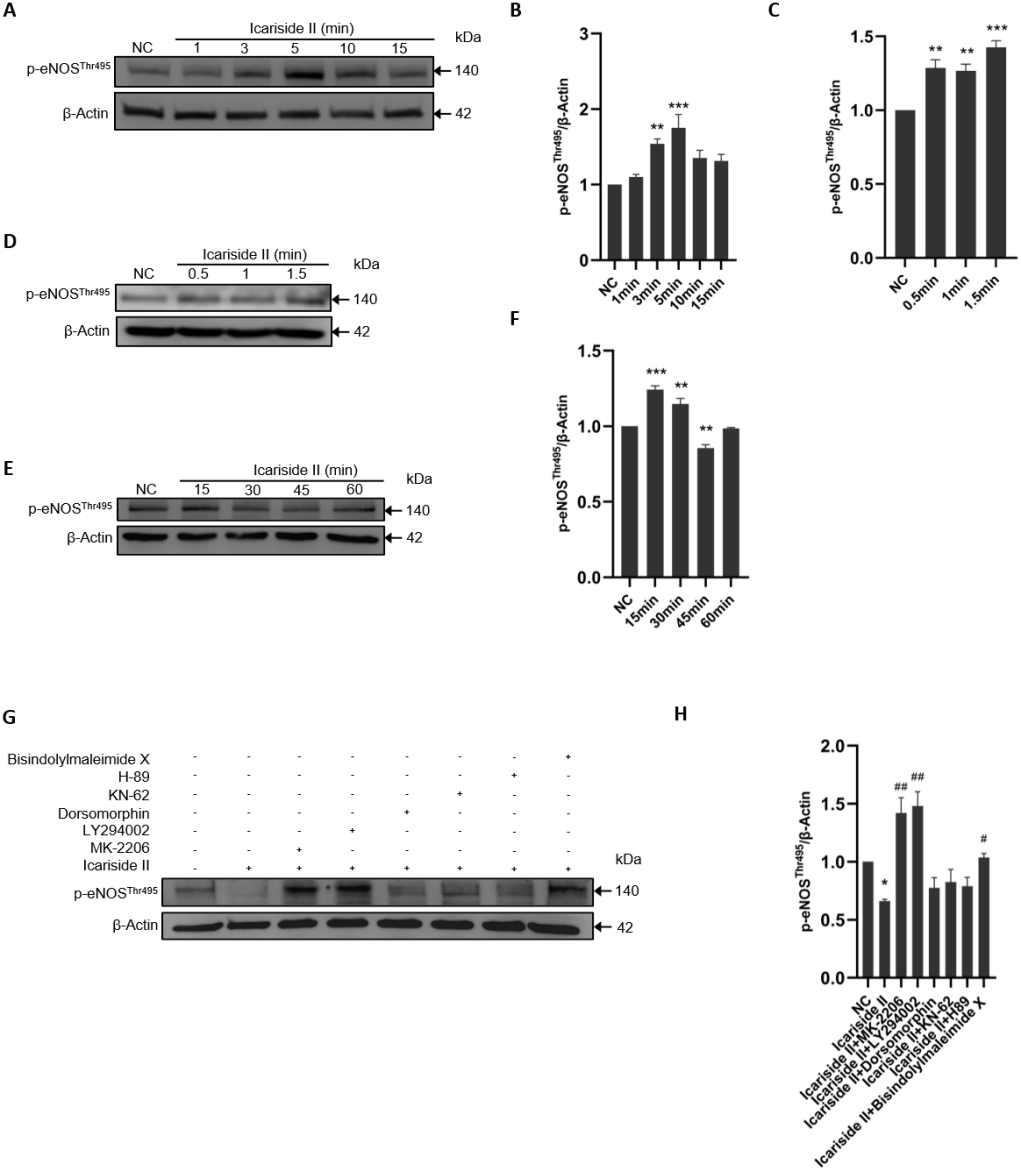
Effect of Icariside II on the expression of p-eNOS^Ser1177^ via PI3K/AKT, AMPK, and PKC signaling pathways. (A, D, E) HUVECs were stimulated with Icariside II for 0.5. 1, 1.5, 3, 5, 10, 15, 30, 45 and 60 min. Western blot was used to analyze the expression of p-eNOS^Thr495^. (B, C, F) Quantitative analysis of p-eNOS^Thr495^ expression. (One-way ANOVA:^∗∗^ *p* < 0.01,^∗∗∗^ *p* < 0.001, experimental groups vs NC). (G) Icariside II-induced eNOS-Thr495 dephosphorylation was abrogated by PI3K, AKT, and PKC inhibitors (LY-294002, MK-2206, and Dorsomorphin), while other inhibitors were not affected. (H) Quantitative analysis of p-eNOS^Thr495^ expression. (One-way ANOVA: ^∗^ *p* < 0.05, experimental groups vs NC, # *p* < 0.05, ## *p* < 0.01, experimental groups vs Icariside II group). Results are shown as one representative blot and as the mean ± SEM of quantified data from three independent experiments.

To study the effect of Icariside II on the phosphorylation of NOS-Thr495, HUVECs were stimulated with Icariside II (10^−6^ M) for 0, 1, 3, 5, 10, and 15 min. The expression level of p-eNOS^Thr495^ was higher in Icariside II treated group compared with the normal control group and peaked at 10 min (Figs. 7A, 7B). As a negative regulatory residue, the up-regulation of eNOS-Thr495 phosphorylation was often associated with the decrease of NO release which was not in accordance with the results above. To investigate whether Icariside II can impact the rapid dephosphorylation of eNOS-Thr495, Icariside II was applied for stimulating HUVECs for multiple durations (0, 0.5, 1, 1.5, 15, 30, 45, and 60 min). The results showed that the phosphorylation of eNOS-Thr495 was significantly increased only 0.5 min after Icariside II stimulation, and lasted to 30 min (Figs. 7C–7F). Icariside II showed a significant decrease on the expression of p-eNOS^Thr495^ after 45-minutes stimulation, which was not found at the time-point of 60 min (Figs. 7E, 7F).

To clarify the specific mechanism involved in eNOS-Thr495 dephosphorylation, 45 min was selected as the stimulation time of Icariside II. The results showed that PI3K inhibitor (LY294002), AKT inhibitor (MK-2206), and PKC inhibitor (Bisindolylmaleimide X) significantly increased the expression level of p-eNOS^Thr495^ down-regulated by Icariside II. In contrast, AMPK inhibitor (Dorsomorphin), CaMKII inhibitor (KN-62), and PKA inhibitor (H-89) did not show similar effects on eNOS-Thr495 (Figs. 7G, 7H). It suggested that Icariside II could rapidly regulate the dephosphorylation of eNOS-Thr495 via activating PI3K/AKT and PKC signaling pathways.

### Icariside II did not influence eNOS-Ser113 phosphorylation

**Figure 8:**
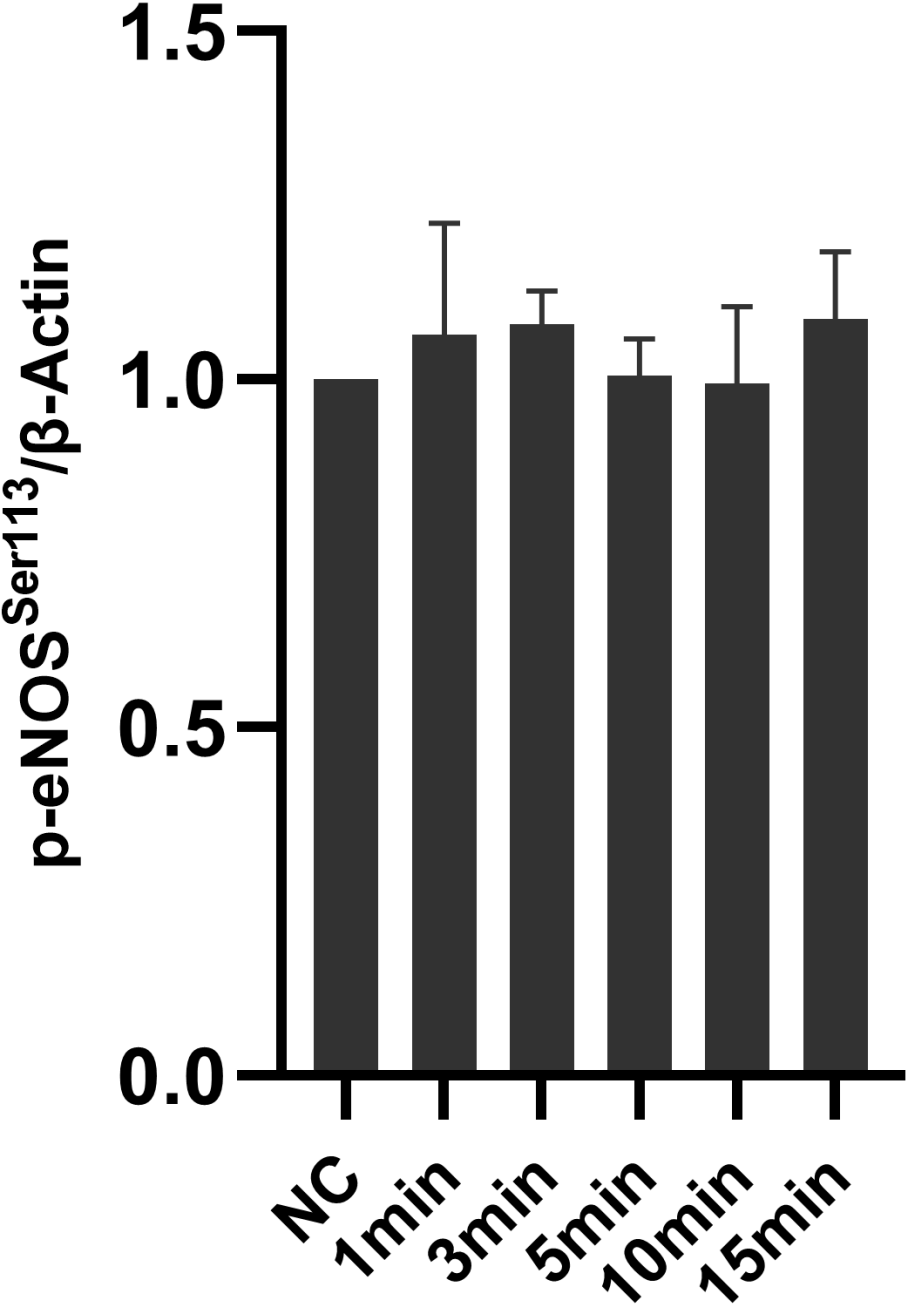
Effect of Icariside II on the expression of p-eNOS^Ser113^. HUVECs were stimulated with Icariside II for 1, 3, 5, 10, and 15 min. Quantitative analysis of p-eNOS^Ser113^ expression. (One-way ANOVA:^∗^ *p* < 0.05,^∗∗^ *p* < 0.01,^∗∗∗^ *p* < 0.001, experimental groups vs NC). Results are shown as the mean ± SEM of quantified data from three independent experiments.

To research the effect of Icariside II on eNOS-Ser113 phosphorylation, time-points of Icariside II were chosen as the same as before (0, 1, 3, 5, 10, and 15 min). No significant differences in the expression of p-eNOS^Ser113^ between Icariside II stimulated groups and the normal control groups (Fig. 8) In brief, the NO release of HUVECs might be affected by Icariside II via regulating the phosphorylation of eNOS-Ser1177 and eNOS-Thr495, not eNOS-Ser113.

## Discussion

**Figure 9:**
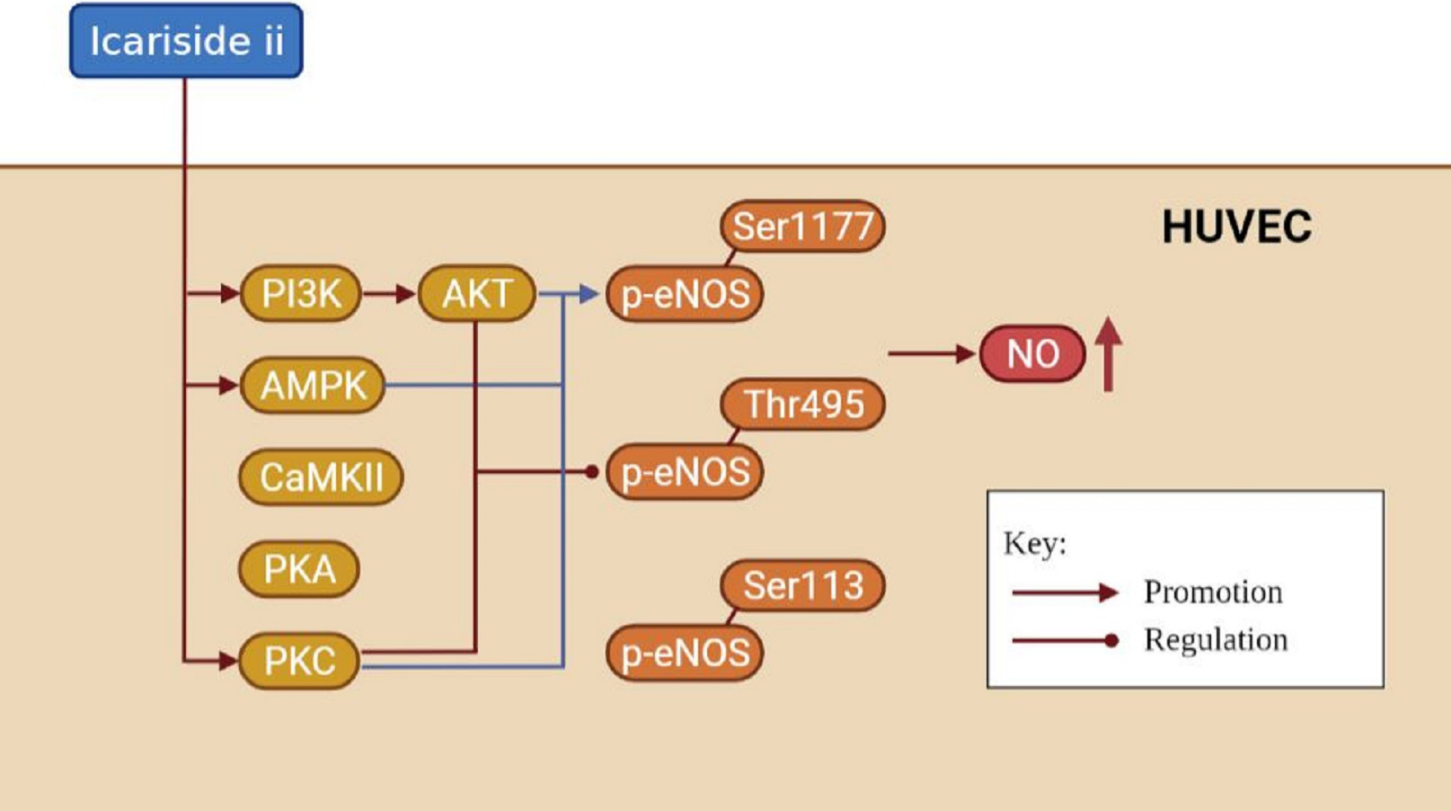
The mechanism of increased NO production affected by Icariside II. Icariside II is able to promote rapid eNOS-Ser1177 phosphorylation by activating PI3K/AKT, AMPK, and PKC signaling pathways, while regulating rapid eNOS-Thr495 dephosphorylation/phosphorylation by activating PI3K/AKT and PKC signaling pathways, thereby up-regulating eNOS activity, and in turn increase NO release. Abbreviation: AKT, Protein kinase B; PI3K, Phosphatidylinositol-3-kinase; AMPK, AMP-activated protein kinase; CaMKII, Calcium-CaM-dependent protein kinase II; PKA, Protein kinase A; PKC, Protein kinase C.

It is well known that the NO released by eNOS is essential for endothelial cell function, which can be regulated by eNOS phosphorylation (Goshi, Zhou & He, 2019; Mount, Kemp & Power, 2007). Endothelial function is closely related to cardiovascular diseases, andrology diseases, kidney diseases, etc (Rajendran et al., 2013; Jourde-Chiche et al., 2019). In this paper, we found that Icariside II rapidly induced the phosphorylation of eNOS-Ser1177 and eNOS-Thr495 via multiple signaling pathways, and rapidly increased NO release in HUVECs, demonstrating the great potential of Icariside II in the treatment of multiple diseases (Fig. 9).

In this study, Icariside II was found to promote the eNOS-Ser1177 phosphorylation of HUVECs in 15 min. The phosphorylation of eNOS-Ser1177 may disrupt the autoinhibitory function of the eNOS carboxy-terminus, thereby rendering the activation of eNOS (Mount, Kemp & Power, 2007; Lane & Gross, 2002). In short, most stimulations that activate eNOS promote phosphorylation of the eNOS-Ser1177, including drugs (such as shenfu injection and atorvastatin) (Zhu et al., 2020; Manickavasagam et al., 2007), compounds (such as betulinic acid and Propionyl-l-carnitine) (Jin et al., 2016; Ning & Zhao, 2013), mechanical factors (Ghimire et al., 2019; Balligand, Feron & Dessy, 2009), and humoral factors (Pooja et al., 2018), and ultimately lead to increase NO synthesis. Li et al. (2015) indicated that Icariside II could increase p-eNOS^Ser1177^ expression of human cavernous endothelial cells down-regulated by high-glucose conditions in 4 days. Few studies researched the effect of Icariside II on eNOS-Ser1177 phosphorylation within a short time (less than 1 h). The phosphorylation of eNOS-Thr495 in the Ca2+/CaM binding domain could reduce the activity of eNOS and decrease the NO release of HUVECs (Mount, Kemp & Power, 2007). We found that Icariside II was able to increase the expression of p-eNOS^Thr495^ within 30 min and decrease it at 45 min. Evidence suggested that dephosphorylation of eNOS-Thr495 was coordinated with the activation of eNOS-Ser1177 phosphorylation (Harris et al., 2001; Michell et al., 2001; Fleming et al., 2001; Peluso et al., 2018), while it had also been reports of the opposite result (Schmitt et al., 2009).

Notably, a significant increase in NO release of HUVECs was found after Icariside II stimulation for less than 60 min, which may be associated with rapidly up-regulated eNOS-Ser1177 phosphorylation. However, the increased eNOS-Thr495 phosphorylation was also observed during the same period of Icariside II stimulation (less than 30 min), which was inconsistent with the result of up-regulated NO release. It might be caused by the fact that the up-regulated phosphorylation of eNOS-Ser1177 played a dominant role and may override the negative effect of eNOS-Thr495 phosphorylation. In addition, although eNOS-Thr495 was indicated to be the negative regulatory residue, after mutation of eNOS-Thr495 to alanine and mimicking the dephosphorylation of eNOS-Thr495, Lin et al. (2003) found the occurrence of “uncoupling” eNOS, which was often associated with the down-regulation of the NO release. However, more research is needed to give more powerful evidence for these hypotheses, such as the measurement of ROS. Even so, we found significant dephosphorylation of eNOS-Thr495 at 45 min, which makes it easier to explain why the NO release in HUVECs was increased after Icariside II stimulation.

Different from eNOS-Ser1177 and eNOS-Thr495, only a handful of studies focused on eNOS-Ser113, phosphorylation of which was usually considered to inhibit the activation of eNOS. It was reported that cyclin-dependent kinase 5 was able to up-regulate eNOS-Ser113 phosphorylation, decrease eNOS dimer stability, and reduce NO release (Lee et al., 2010). However, Urano et al. (2008) found that angiopoietin-related growth factors could activate the ERK1/2 signaling pathway in HUVECs and increase the phosphorylation of eNOS-1177 and eNOS-Ser113, so as to up-regulate the production of NO. However, results in this study did not indicate a significant effect of Icariside II on eNOS-Ser113 phosphorylation. In short, the effect of Icariside II on eNOS phosphorylation is complex which promotes the NO release of HUVECs and demonstrates the potential of Icariside II to regulate endothelial function.

Although Icariside II stimulation was reported to increase the expression of total eNOS in endothelial cells at a late time (48 or 96 h) (Liu et al., 2011; Li et al., 2015), no changes in total eNOS were observed at an early time (less than 1 h in this study) which may be related to the insufficient time for eNOS transcription and translation.

In this study, the common upstream signaling pathways of eNOS phosphorylation were detected by western blot, including PI3K, AKT, AMPK, CaMKII, PKA, and PKC[21-25]. The results indicated that Icariside II was able to activate the eNOS-Ser1177 phosphorylation via P13K/AKT, AMPK, and PKC significant pathways, and the eNOS-Thr495 dephosphorylation by P13K/AKT, and PKC signaling pathways. PI3K/AKT and AMPK signaling pathways may be the positive regulator of eNOS which play an important role in endothelial cell survival, mobilization, migration and homing (Chu et al., 2017; Rodríguez et al., 2021). It was reported that the activation of AMPK/PI3K/AKT signaling pathway could increase eNOS-Ser1177 phosphorylation and decrease eNOS-Thr495 phosphorylation (Xing et al., 2015). In this study, the activation of AMPK signaling pathway was not associated with the eNOS-Thr495 dephosphorylation. Signorello et al. (2009) suggested that homocysteine was able to stimulated the eNOS-Thr495 phosphorylation and the dephosphorylation of eNOS-Ser1177 by PKC activation which was inconsistent with the results in this study.

It was worth noting that the effect of Icariside II on eNOS phosphorylation was only researched in less than 1 h, the effect over a longer period needs to be explored in more experiments. It was reported that compound 21 and quercetin were also able to regulate the eNOS phosphorylation rapidly (Peluso et al., 2018; Li et al., 2012). Because of the short stimulation time (less than 15 min), the total signaling pathway proteins (Such as PI3K, AKT, AMPK, and PKC) were not detected in this study. Several signaling pathway inhibitors were applied in this study, some of which (such as dorsomorphin and LY294002) were often limited by off-target effects (Hao et al., 2010; Kumar et al., 2008). Further lines of research would be necessary to complete the data on the expression of the total signaling pathway proteins and to solve the problem of off-target effects of inhibitors. Flavonoids are widely distributed by plants and have multiple potential biological benefits, including regulating endothelial function, anti-inflammatory, anti-cancer, anti-fungal, etc (Zakaryan et al., 2017). As one of the active flavonoids, Icariside II can regulate eNOS phosphorylation rapidly which suggests that Icariside II may assist in the treatment of acute diseases (such as myocardial infarction and cerebral ischemia) and erectile dysfunction which has been preliminary explored in other *in vivo* and *in vitro* studies (Hu et al., 2020; Gao et al., 2020; Liu et al., 2020; Gu et al., 2021; Xu et al., 2015). Although this study provides important knowledge to the field of eNOS phosphorylation and the physiological and pharmacological effect of Icariside II, whether the results of our *in vitro* study are consistent with the *in vivo* situation still needs to be verified.

## Conclusions

Our study found that Icariside II could regulate rapid phosphorylation of eNOS-Ser1177 and eNOS-Thr495 via multiple signaling pathways and promote the NO release of HUVECs, regulating endothelial function in a short time. It may provide a novel pharmacologic molecule to assist in the treatment of several diseases. More investigations are required to explore the therapeutic potential of Icariside II.

##  Supplemental Information

10.7717/peerj.14192/supp-1 Supplemental Information 1 Original Images for Blots and Gels

## Additional Information and Declarations

### Data Availability

The following information was supplied regarding data availability: The original images for blots and gels are available in the Supplementary File.
